# The New Pharmacopœia

**Published:** 1898-01-15

**Authors:** 


### EDITOR'S LETTER-BOX.

[Our Correspondents are reminded that prolixity is a great bar to publication, and that brevity of style and conciseness of statement greatly facilitate early insertion.]

### THE NEW PHARMACOPŒIA.

DR. NESTOR TIRARD writes: As Secretary of the Pharmacopœia Committee of the General Medical Council, I am instructed to forward the enclosed to you for publication. This enclosure has also been forwarded to the various medical and pharmaceutical journals.

74, Harley Street, W., January 11th, 1898.

"With reference to an article in the *Chemist and Druggist* of January 8th, 1898, purporting to publish the first part of a critical review of the forthcoming British Pharmacopœia, we are officially informed that such publication is entirely unauthorised by the General Medical Council, and that criticism at present is not possible because the work is still incomplete.

"We are also officially informed that the article already published contains numerous inaccuracies, and that it bears internal evidence of having been based upon an early unrevised proof."

